# Clinical and pathological characteristics in 214 Danish weaners euthanized because of umbilical outpouchings

**DOI:** 10.1186/s40813-024-00401-w

**Published:** 2024-11-22

**Authors:** Marie-Louise Hansen, Inge Larsen, Tina Birk Jensen, Charlotte Sonne Kristensen, Ken Steen Pedersen

**Affiliations:** 1 Department of Veterinary and Animal Sciences, Faculty of Health and Medical Sciences, University of Copenhagen, Grønnegårdsvej 2, 1870 Frederiksberg C, Denmark; 2 Danish Pig Research Centre, SEGES, Agro Food Park 15, 8200 Aarhus, Denmark; 3 Næstved, Denmark

**Keywords:** Umbilical outpouching, Pig, Hernia, Weaner, Welfare, Sustainability, Pathology

## Abstract

**Background:**

Umbilical outpouchings (UO) are common in Danish weaners. In slaughter pigs UOs consist of various pathological diagnoses; however, no studies have assessed the pathology in weaners from randomly selected herds, nor the agreement between clinical examination findings and post-mortem results. The primary objective was to estimate the prevalence of UO-related clinical findings in weaners before euthanasia and the pathologic macroscopic findings after euthanasia. A secondary objective was to assess the agreement between the size of the UO, the presence of ulcers, and the diagnosis before and after euthanasia. Pigs were selected for euthanasia and included in the study because the individual farmer believed the pigs were already unfit for transport, would not make it to slaughter, or would become unsellable.

**Results:**

In total 214 weaners euthanized due to UOs were examined both clinically and post-mortem. Clinically 65.4% of the UOs were large (≥ 11 cm) and 52.3% were unreducible. In the autopsy 78.5% of the UOs were large, and 54.2% had ulcers on their UO. The most prevalent pathological diagnoses were hernia (36.4%), hernia combined with cysts/abscesses (30.8%), cysts (11.7%), and abscesses (11.2%). Adhesions were found in 32.7%, haemorrhage in 22.7%, incarcerated intestines in 8.9%, and connective tissue related to the UO in 51.9% of the pigs post-mortem. The agreement between the size of the UO in the clinical examination and post-mortem was good, as was the sensitivity and specificity for detecting ulcers in the clinical examination compared to post-mortem.

**Conclusion:**

Hernias were the most common pathological diagnosis in weaners euthanized due to UOs, and many pigs had complications related to their UO such as ulcers, adhesions, haemorrhage, or incarcerated intestines—all conditions that could impact the pig’s welfare. The presence of connective tissue in the UO in more than half of the pigs indicated that many of the UOs had been present for an extended period. Clinical identification of the umbilical ring is difficult, but the clinical examination is useful for determining the size of the UO and the presence or absence of an ulcer, both critical factors when assessing a pig’s welfare and fitness for transport. Clinical examination, however, has limited value in determining the aetiology of UOs.

**Supplementary Information:**

The online version contains supplementary material available at 10.1186/s40813-024-00401-w.

## Background

Umbilical outpouchings (UO) are common in Danish pigs and affect their welfare. A cross-sectional study reported a UO prevalence of 2.9% in Danish weaners [1] corresponding to approximately a million UO pigs yearly; pigs that need appropriate handling, such as housing in hospital pens and on soft bedding or timely euthanasia if the UO becomes too large or starts to ulcerate. Danish legislation deems pigs with large (6–10 cm in 15–35 kg pigs, > 15 cm in finishers) or ulcerated UOs unfit for transport and if the pig has affected general condition, reduced growth, or obvious impeded movement it is to be euthanised immediately [2].

Today a high proportion of pigs in conventional Danish herds receive metaphylactic antibiotics at birth to prevent omphalitis and subsequently UOs, sepsis, and limb infections [1]. However, research presents conflicting evidence on the effects of antibiotic treatment in preventing UO [3–7]. Knowing the aetiology behind the UOs is relevant when investigating preventive measures as surgery is the only effective treatment currently available. Lege artis surgery is however too expensive to perform in Denmark and the Danish Animal Welfare Council deems alternatives such as elastrators unacceptable because an elastrator on the UO induces ischaemic necrosis and pain; which is unacceptable from a welfare point-of-view [8].

Sustainable pig production requires that as many pigs as possible reach the abattoir. Pigs euthanized before slaughter are a waste of pig lives, work effort, and money. At present, there are no peer-reviewed studies of weaners euthanized because of UOs on random farms. Therefore, it is important to investigate whether UO weaners euthanized on farms present similar diagnoses as pigs slaughtered with UO or if they constitute a different subtype of UOs, thereby requiring different preventive measures.

To increase the number of pigs sold/handled/transported at 30 kg or delivered to slaughter in Denmark, pigs with UOs can be examined by a herd veterinarian and receive a veterinary certificate stating that the pig is fit for transport under certain conditions. Sometimes disagreements arise between pigs stated fit by the herd veterinarian and the official veterinarian at the abattoir, which may occur because of different conclusions based on clinical and pathological examinations. Pigs with UOs having different pathological diagnoses may differ concerning fitness for transport. Therefore, it is beneficial to know the agreement between clinical and pathological examinations. In the herd, assessment of welfare, prognosis, and/or relevance of surgery also makes it relevant to know the agreement between clinical and pathological assessment of UOs, including if it is an uncomplicated hernia and how large the umbilical ring is. To our knowledge, no one has published how well findings in a clinical examination agree with the pathological findings during autopsies.

This study aimed at characterizing the UO of weaners euthanized on farms. The study included pigs that were euthanized either because the farmer did not believe in their current/future ability to be fit for transport or because of a poor prognosis for survival. In Denmark there are no regulations prohibiting the euthanasia of an animal, that is solely the decision of the owner, therefore euthanasia of production animals is often the treatment of choice since the economic value of a single pig is low.

The primary objective was to estimate the prevalence of UO-related clinical findings in weaners before euthanasia and the pathologic macroscopic findings after euthanasia. A second objective was to assess the agreement between the clinical findings in UO in weaners before euthanasia to the pathologic macroscopic findings after euthanasia.

## Results

A total of 214 weaners were clinically examined while alive and autopsied post-mortem. The pigs originated from 29 herds, and the number of pigs per herd varied from 3 to 17. The information about the weight, body condition, and sex of the weaners is presented in Table 1.

**Table 1:** Results from the clinical examination of 214 weaners selected for euthanasia because of UO

Variable	Level	N	%
Sex	Male	81	37.9
	Female	133	62.1
Size UO^a^	Small 4–7 cm	17	7.9
	Medium 8–10 cm	57	26.6
	Large ≥ 11 cm	140	65.4
Reducibility	Yes	45	21.0
	Partly	54	25.2
	No	112	52.3
	NA^b^	3	1.4
Texture	Soft	89	41.6
	Mix	26	12.1
	Hard	96	44.9
	NA	3	1.4
Ulcer	Yes	95	44.4
	No	118	55.1
	NA	1	0.5
Ulcer size^c^	Small 2–3 cm	11	11.5^d^
	Medium 4–7 cm	53	55.2
	Large ≥ 8 cm	29	30.2
	NA	3	3.1
Umbilical ring	Yes	83	38.8
	No	128	59.8
	NA	3	1.4
Size pig	< 10 kg	21	9.8
	10–20 kg	87	40.7
	> 20 kg	105	49.1
	NA	1	0.5
Body condition score	Normal	181	84.6
	Affected	31	14.5
	Emaciated	2	0.9
UO classification	Uncomplicated hernia^e^	40	18.7
	Non-reducible UO^f^	171	79.9
	NA	3	1.4

^a^Sum of height and width in cm

^b^Not available

^c^Sum of length and width in cm

^d^Denominator = 96 for all ulcer sizes

^e^Fully reducible outpouching and soft in texture

^f^A hernia ring might still be recorded, but the outpouching had to be fully reducible to be recorded as uncomplicated hernia

### Clinical findings before euthanasia

Most of the UOs were categorised as large, and ulcers were recorded on approximately half of the UOs, most of the ulcers being categorised as medium or large.

An umbilical ring could be palpated in 38.8% of the UOs, but only 18.7% were categorized as uncomplicated hernias (soft and fully reducible). The mean diameter of the umbilical rings was 2.9 cm ranging from 0.5 to 7 cm with a median diameter of 3 cm.

Table 1 shows the clinical findings in detail.

### Macroscopic findings during autopsy

At autopsy, most of the UOs were categorized as large, and ulcers were found on more than half of the UOs. Most of the ulcers were, in contrast to the clinical examination, classified as small or medium. The most prevalent pathological diagnoses were hernia (36.4%) or hernia in combination with cysts/abscesses (30.8%). Approximately 1/3 of the pigs had abscesses alone or in combination with hernias or cysts.

Among the autopsied pigs an umbilical ring was found in 83.2% with a mean diameter of 2.7 cm ranging from 0.5 to 10 cm and a median diameter of 2 cm. Many pigs also experienced complications related to their UO in the form of connective tissue, adhesions,^1^ inflammation of the umbilical structures, haemorrhage, or even incarcerated intestines. The mean thickness of the connective tissue related to the UO was 14.2 mm, ranging from 1 to 50 mm with a median of 10 mm.

Almost 10% of the pigs displayed varying degrees of incarcerated intestines, with the ileum being the most frequently affected segment. Moreover, intestinal diverticula were observed on the antimesenteric border of 2.8% of the pigs (6 out of 214 pigs). Of these, 83.3% (5 of 6 pigs) were located on the ileum, while one diverticulum was situated on the jejunum. Table 2 shows the results from the autopsies.

**Table 2:** Results from autopsies of 214 weaners euthanised because of UOs

Variable	Level	N	%
Size UO	Small 4–7 cm	9	4.2
	Medium 8–10 cm	36	16.8
	Large ≥ 11 cm	168	78.5
	NA^a^	1	0.5
Ulcer	Yes	116	54.2
	No	97	45.3
	NA	1	0.5
Ulcer size	Small 2–3 cm	49	41.8^b^
	Medium 4–7 cm	48	41.0
	Large ≥ 8 cm	19	16.2
	NA	1	0.9
Umbilical ring	Yes	178	83.2
	No	30	14.0
	NA	6	2.8
Pathological diagnoses	Hernia	78	36.5
	Hernia + cyst	40	18.7
	Hernia + abscess	26	12.1
	Hernia + cyst + abscess	6	2.8
	Cyst	25	11.7
	Abscess	24	11.2
	Cyst + abscess	6	2.8
	Paddle formed proliferation	0	0
	Other diagnoses^c^	9	4.2
Connective tissue^d^	Present	111	51.9
Adhesions^e^	Present	70	32.7
Omphaloarteritis	Present	7	3.3
Omphalophlebitis	Present	19	8.9
Urachitis	Present	9	4.2
Ligaments	None	48	22.4
	Ligamentum teres hepatis falciforme	64	29.9
	Ligamentum umbilicale medianum	34	15.9
	Ligamentum teres hepatis falciforme + Ligamentum umbilicale medianum	67	31.3
	NA	1	0.5
Urachus^f^	Patent	32	15.0
	Persistent	2	0.9
Haemorrhage^g^	Present	49	22.9

Examples of diagnoses can be seen in additional material—*Pathological diagnoses of umbilical outpouchings in pigs: Examples from the present data sampling*

^a^Not available

^b^Denominator = 117 for all ulcer sizes

^c^One of these pigs had persistent urachus, three had fibrotic tissue combined with adhesions, three pigs had fibrotic tissue alone, one had a haematoma, and one was undiagnosed. Eight of the nine pigs had ulcers

^d^In relation to the UO

^e^Either between intestines or from intestines to the UO

^f^Patent = lumen can still be found. Persistent = urine is leaking through the umbilicus

^g^Blood clots in the abdomen or UO or as bleeding in the wall of the UO or the intestines

### Agreements between clinical examination and autopsy results

#### Agreement for assessment of UO size at clinical examinations and autopsies

In 76.2% (CI^2^ [0.7–0.82]) of the autopsied pigs the size of the UO was recorded the same size category as in the clinical examination, whereas 18.7% (CI [0.14–0.25]) were a size category larger, and 3.7% (CI [0.02–0.07]) were a size category smaller. Information was missing for one pig. In 1% (CI [0.001–0.03]) the UOs changed more than one category, from small in the clinical exam to large in the autopsy.

#### Agreement for assessment of ulcers on the UO at clinical examinations and autopsies

Table 3 shows the background for calculating the sensitivity and specificity for ulcer detection in the clinical examination and using the ulcers found during autopsies as the reference standard.

**Table 3:** Agreement between clinical and autopsy examination for ulcer detection in pigs with UOs

	Ulcer autopsy (reference)			
	Yes	No	*NA*	
Ulcer clinical exam (test)				
Yes	86	8	*1*	94
No	30	88		118
*NA*		*1*		
	116	96		212^a^

^a^Two pigs had NA values for either the clinical examination or at the autopsy

The sensitivity was 74.1% (86/116) and the specificity was 91.7% (88/96). The 30 (14%) ulcers missed in the clinical examination were either size small or medium.

#### Agreement for assessment of umbilical rings at clinical examinations and autopsies

Of the 83 pigs with records of umbilical rings in the clinical examination, 6 pigs (7.2% (CI [0.03–0.15]) did not have an umbilical ring at autopsy. Among the 77 pigs with umbilical rings at both clinical and autopsy examination, 44.5% (CI [0.34–0.56]) had the same size in the autopsy, 7.2% (CI [0.03–0.15]) had smaller rings and 41% (CI [0.30–0.52]) had larger rings.

Of the 128 pigs with no record of umbilical rings in the clinical examination, 72.7% (CI [0.64–0.80]) did have hernia rings of varying sizes. In 23.4% (CI [0.16–0.32])) of the pigs no umbilical ring was demonstrated in either the clinical examination or the autopsy.

In total 52.2% (CI [0.45–0.6])) of the rings found during the autopsies were missed in the clinical examination; 76.3% (CI [0.66–0.85]) of these missed rings were small 0.5–3 cm, and 20.4% (CI [0.13–0.30]) were rings larger than 3 cm. Adhesions were found in 20.0% (CI [0.07–0.41]) of the smallest rings (0.5–1 cm), in 63.0% (CI [0.48–0.77]) of rings 1.5–3 cm, and in 26.3% (CI [0.09–0.51]) of rings larger than 3 cm. Intestines/oment/cysts or abscesses also obliterated some umbilical rings, and the sheer size of the UO prevented the reduction of its contents to the abdomen in some pigs, making the ring difficult to feel. Several of the cysts had umbilical rings, without intestinal content, either because the ring was small or because the cyst constituted the entire UO, and some hernias have no registrations of umbilical rings because of adhesions between UO and intestines/oment.

#### Agreement for assessment of UO type at clinical examinations and autopsies

Of the 40 pigs diagnosed with an uncomplicated hernia at the clinical examination 82.5% (CI [0.67–0.93]) were diagnosed with hernia at the autopsy, 10% (CI [0.03–0.24]) were diagnosed with a hernia in combination with cyst/abscess or both, and 5% (CI [0.01–0.17]) were diagnosed with cysts without hernia, and one pig were diagnosed with fibrosis.

Of the 171 pigs diagnosed with a non-reducible UO at the clinical examination 25.7% (CI [0.19–0.33) were diagnosed with a hernia at the autopsy, 39.2% (CI [0.32–0.47]) were diagnosed with a hernia in combination with cyst/abscess or both, 12.9% (CI [0.08–0.19]) were diagnosed with cysts without hernia and 14.0% (CI [0.09–0.20]) were diagnosed with abscesses, 3.5% (CI [0.01–0.07]) were diagnosed with cyst/abscess combination, 2.9% (CI [0.01–0.07] had fibrosis and/or adhesions and 1.8% (CI [0.001–0.05]) had other diagnoses.

The sensitivity for detecting uncomplicated hernias was 42.9% (33/77) whereas the specificity was 94.8% (127/134) as shown in Table 4.

**Table 4:** Agreement between clinical and autopsy examination for diagnosing uncomplicated hernias clinically in pigs with UOs

	Diagnose autopsy (reference)		
	Hernia	UO	
Clinical exam (test)			
UH	33	7	40
UO	44	127	171
*NA*	1	*2*	
	77	134	211^a^

*UH* Uncomplicated hernia

^a^3 pigs had missing values for either reducibility or texture in the clinical examination

## Discussion

In this study, hernia was the most prevalent condition in weaners with UO, and the prevalence of intra-abdominal lesions in the form of haemorrhages, adhesions, and incarcerations was high, despite the examined pigs showing limited outer signs of unthriftiness. Hernia also dominates in other studies [9, 10], even though they are conducted on other age groups and with other inclusion criteria. Ulcers on more than half of the UOs are a concern from a welfare point-of-view, as well as the finding of haemorrhage, adhesions, and incarcerations; all conditions that probably influence the welfare of the affected pigs negatively. To the authors' knowledge, there is no peer-reviewed research on how painful UOs are in pigs, but pain is described in humans with umbilical hernias [11, 12], and until proven otherwise the same should be expected in pigs. Ulcers on the UO will probably also cause pain as is seen in e.g. shoulder ulcers in sows [13].

The finding of connective tissue in more than half of the pigs shows that most of the UOs have been present for a longer period [14], which increases the risk of compromised welfare.

This study found a lower prevalence of cysts and a higher level of diagnoses with abscesses compared to two Danish studies examining slaughter pigs who found ~ 70% hernia and ~ 30% cysts [10, 15]. The newest study on slaughter pigs with ulcerated UOs finds no abscesses, which could be caused by their decision to exclude pigs with hard UOs [10]. Since most of the weaners in this study were euthanized because they had large UOs and maybe ulcers (and as such were unfit for transport) they differed from the UO pigs who make it to slaughter.

Hovmand-Hansen et al. [9] also autopsied weaners and reported findings like this study; Hernias being the most prevalent and abscesses alone or in combination with other diagnoses in a quarter of the UOs. The number of ligaments recorded in the two studies differed more, mainly because lig. umbilicale medianum was found in more pigs in this study. The herds Hovmand-Hansen examined were chosen based on a history of UO problems, so might not be representative of other herds concerning aetiologies. Whether the level of inflamed umbilical structures or the presence of ligaments is the same in these UO pigs, as in normal pigs, is unknown since no such studies on normal pigs exist. We hypothesize that problems with the regression of the umbilical structures might keep the umbilical ring open and contribute to the large number of hernias found.

None of the other Danish studies reported findings of intestinal diverticula. Some of the diverticula seen in this study are probably Meckel’s diverticula. Meckel's diverticula are situated at the ileum and result from improper regression of the proximal portion of the vitelline duct^3^ [14].

Agreements between size categories in the clinical examination compared to the autopsy results were good, most of the pigs were in the same category and if they were not, most were only one size category off. A UO being smaller in the autopsy than the clinical examination is very common because the pig is placed in dorsal recumbency and content might therefore disappear inside the abdomen where the pressure is now reduced, this is often the case especially if the umbilical ring is big. The size of the UO is a relevant parameter when transporting pigs in Denmark [2] and using a ruler to measure the size of the UO in live pigs makes it possible to obtain precise results.

The agreement between ulcers found in the clinical examination and the autopsies is also relatively good with a sensitivity of 74.1% in this study, and a specificity of more than 90%. The examination is somewhat subjective, however, with clean pigs, it should be possible to obtain sensitivities and specificities for ulcer detection of close to 100%, whereas manure and faeces on the pigs, make it difficult to see and feel ulcers. Discrepancies in this study, between the clinical examination and autopsies, where the clinical examination misses some ulcers, occur because many of the pigs were dirty.

Diagnosing uncomplicated hernias solely based on a clinical examination on standing pigs has a low sensitivity but a very good specificity. Ultrasound is another tool useful for detecting umbilical rings, but not very feasible to use in pig herds since it requires skills and cannot be done by laymen.

This study was nested within a larger prevalence study [1] and the pigs the farmers chose to euthanize in this study differ clinically from the randomly sampled pigs in the same herds^4^: they have larger UOs and a higher prevalence of ulcers, but similar reducibility and texture of the UO; this might contribute to the high level of intra-abdominal lesions, but a post-mortem study on randomly sampled UO weaners is needed to confirm whether intra-abdominal lesions are common in all UO pigs, or if large or ulcerated UOs have a higher level of complications.

## Conclusion

Hernias were the most common pathological diagnosis in weaners euthanized due to UOs, and many pigs had complications related to their UO such as ulcers, adhesions, haemorrhage, or incarcerated intestines—all conditions that could impact the pig´s welfare. The presence of connective tissue in the UO in more than half of the pigs indicated that many of the UOs had been present for an extended period.

Clinical identification of the umbilical ring is difficult, but the clinical examination is useful for determining the size of the UO and the presence or absence of an ulcer, both critical factors when assessing a pig's welfare and fitness for transport. Clinical examination, however, has limited value in determining the aetiology of UOs.

## Methods

### Setting

This cross-sectional study was nested within a larger study examining the prevalence of UOs in piglets and weaners [1]. Twentynine herds selected randomly from the Danish Husbandry Register volunteered to participate. All herds used Danbred or Danish genetics, had at least 200 sows and 800 weaners registered, and were within a 3-h drive from Copenhagen. The pigs were all crossbreds between Landrace/Yorkshire/Duroc [1].

As part of the prevalence study farmers were asked which pigs, they planned to euthanize because of UOs and other possible comorbidities. Pigs in this study originate from the random sample as well as the sick pens examined in the aforementioned study.

Therefore, although pigs in this study comprise a convenience sample, they are considered representative of weaners euthanized due to UOs in random Danish herds.

### Study design

Pigs were included in the study based on the farmer's decisions. Many of the pigs were already unfit for transport due to the size of the UO or the occurrence of ulcers. However, some were euthanized because the farmer believed the pig would later become unsellable/unfit for transport. Criteria for euthanasia vary therefore among the individual farmers and herds; if all pigs were sold for export as weaners the tolerance for euthanising UO pigs was very low, whereas production herds with finishers, had a higher threshold for euthanising UO pigs in the hope of being able to send them for slaughter.

Pigs were euthanised the same day as the clinical examination was performed, to make comparisons between findings on live and dead pigs. All pigs were autopsied in the herd immediately after euthanasia. The first author performed all the clinical examinations as well as the autopsies.

### Clinical examination and autopsies

The clinical examination of live pigs consisted of palpation of the UO with a recording of size, reducibility, ulcers, texture, hernia-ring, and classification of the UO as either an uncomplicated hernia or a UO. The examination was performed on standing pigs following the same procedures and size definitions as Hansen et al. introduced [1]. The pigs were examined standing using a herding board to fixate the pig against the corner of the pen. Using fingers the length and height of the UO and possible ulcers were estimated in cm. The diameter of the umbilical ring was also measured using fingers; if it could be felt with only a fingertip it was recorded as 0.5 cm, and if one or more fingers or the entire hand could be inserted into the umbilical ring, the size was recorded with 0.5 cm intervals until 1.5 cm and in full cm from 2 to 10 cm. The size of the UOs as well as the size of the ulcers were categorised into three categories as shown in Table 5. Table 6 shows the outcome definitions for the various variables recorded in the clinical exam and the autopsy.

**Table 5:** Categorization of UO size and ulcers into categories—small, medium, and large

	Umbilical outpouching category^a^			Ulcer size category		
	Small	Medium	Large	Small	Medium	Large
Weaners	4–7 cm	8–10 cm	≥ 11 cm	2–3 cm	4–7 cm	≥ 8 cm

The UOs are classified into one category based on the sum of their height and width in cm, the same applies to ulcer size where length and width are used [1]

^a^Example: A UO in a weaner measuring 6 times plus 5 cm, sum = 11 → size large

**Table 6:** Definitions of variables used in the clinical examination and the autopsy

Variable	Outcomes	Method	Definition
*Variables related to the pig*			
Body condition score	Normal	Visual	The rib cage was not visible
	Affected		Sunken flanks
	Emaciated		Spine and ribs visible
*Variables related to the UO*			
Reducibility	Yes	Palpation	The UO can be manipulated through the umbilical ring to the abdomen
	Partly		
	No		
Ulcers	Yes	Palpation	Tissue loss of more than 1 × 1 cm
	No		
Uncomplicated hernia	Yes	Palpation	Fully reducible soft outpouching
*Variables related to the autopsy*^a^			
Ligaments	None	Autopsy	No ligaments
	*Ligamentum teres hepatis falciforme*		Ligament from UO to liver
	*Ligamentum umbilicale medianum*		Ligament from UO to bladder
Urachus	Patent	Autopsy	The ligament from the bladder has a lumen
	Persistent		The lumen communicates with the umbilicus
Haemorrhage	Present	Autopsy	Free blood or blood clots in the abdomen or the UO
Pathological diagnoses	Hernia	Autopsy	Only intestinal content in the UO
	Cyst		Fluid-filled caverns in the UO
	Abscess		Pus in the UO
	Paddle formed proliferation		Fibrous-like structure arising from the wall of the hernia or umbilical ring
Adhesions	Present	Autopsy	Adhesions between intestines or omentum to the UO or between intestines alone
*Omphalophlebitis*	Present	Autopsy	Enlargement or pus in the liver vein
*Omphaloarteritis*	Present		Enlargement or pus in the umbilical arteries
*Urachitis*	Present		Enlargement or pus in the urachus

^a^See additional material: *Pathological diagnoses of umbilical outpouchings in pigs: Examples from the present data sampling*

The pigs were euthanized with a stun gun followed by exsanguination.

All pigs were autopsied following this procedure:

1. The pig was placed in dorsal recumbency.
2. The weight category of the pig was estimated visually (< 10 kg, 10–20 kg, > 20 kg).
3. The size of the UO was measured using a ruler (length and height cm)
4. Possible ulcers and their size were recorded, and their size was measured using a ruler (length and width cm).
5. The UO was photographed from the side, front, and back.
6. If a hernia ring was felt the size was recorded in cm.
7. The abdomen was incised circularly on the left side of the UO.
8. Remnants of the foetal circulation were recorded if present (*Ligamentum teres hepatis falciforme/Ligamentum umbilicale medianum*).
9. Other adhesions were noted (e.g. intestinal).
10. Another check for hernia-ring was performed, and if found size was recorded.
11. The UO was palpated, and possible hard areas or swellings were incised. The content was recorded (blood, pus, intestines, serous fluid).
12. Other affected structures were recorded (*omphaloarteritis, omphalophlebitis, urachitis*)

## Supplementary Information

Supplementary Material 1.

## Data Availability

The datasets used and/or analysed during the current study are available from the corresponding author upon reasonable request.

## Footnotes

1 Equivalent to chronic adhesive peritonitis.

2 95% confidence intervals.

3 *Ductus omphalomesentericus*.

4 In the random sample most of the UOs were small or medium size (excluding pigs in sick pens) and ulcers were recorded on 12.5% of the UOs [1].
