# Supplementary material for: Clinical and pathological characteristics in 214 Danish weaners euthanized because of umbilical outpouchings
Source: Porcine Health Manag. 2024 Nov 22;10:54. doi: 10.1186/s40813-024-00401-w (PMC11583573; doi:10.1186/s40813-024-00401-w)
Supplement: Supplementary file 1 — Supplementary Material 1. [file 40813_2024_401_MOESM1_ESM.pdf]

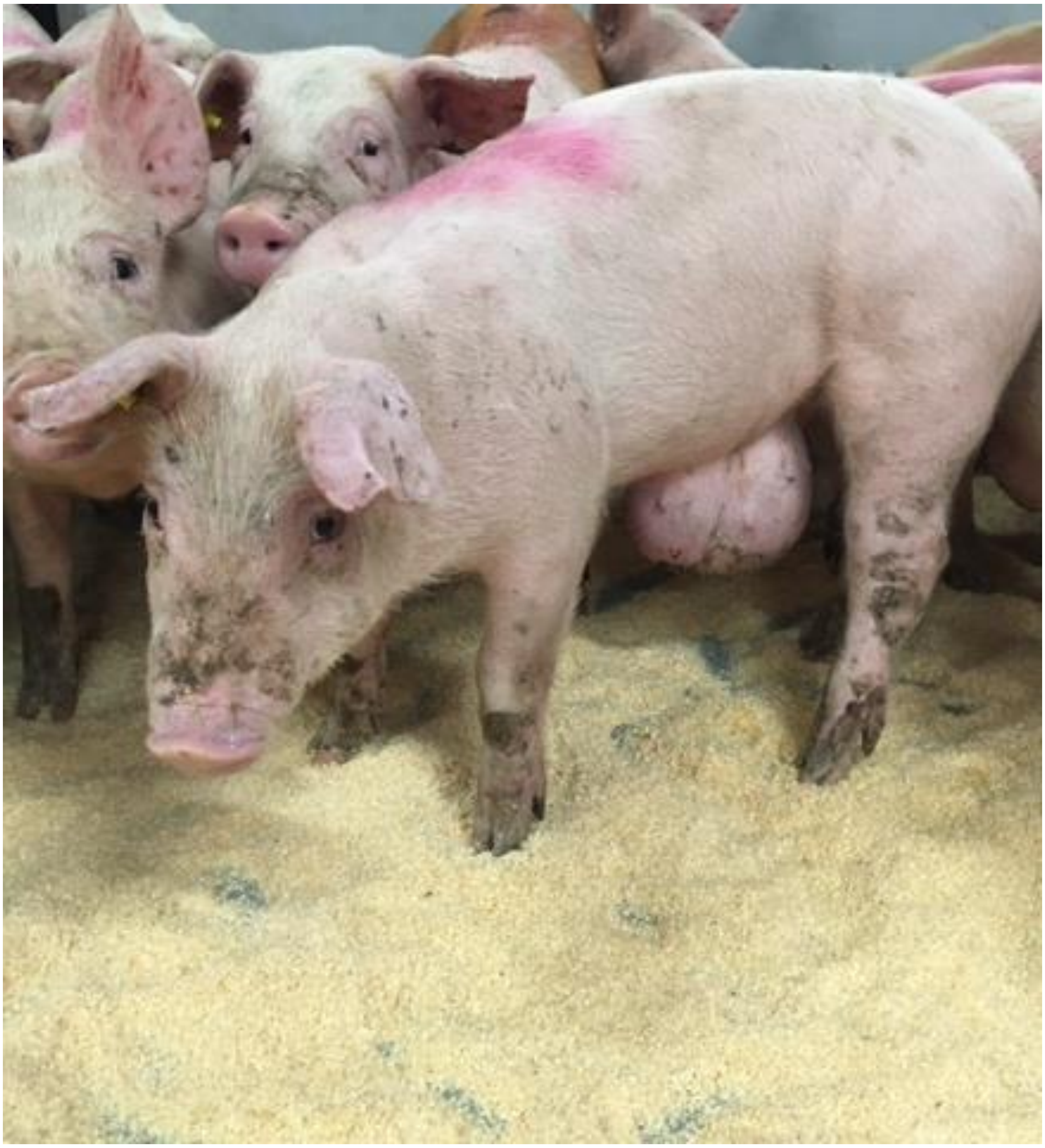

Pathological diagnoses of umbilical outpouchings in pigs: Examples from the present data sampling

Ulcers
Types of umbilical outpouchings
Bleedings/ adhesions
Incarcerated intestines
Normal/ abnormal umbilical ligaments

All pictures are from different pigs unless otherwise indicated

Ulcers

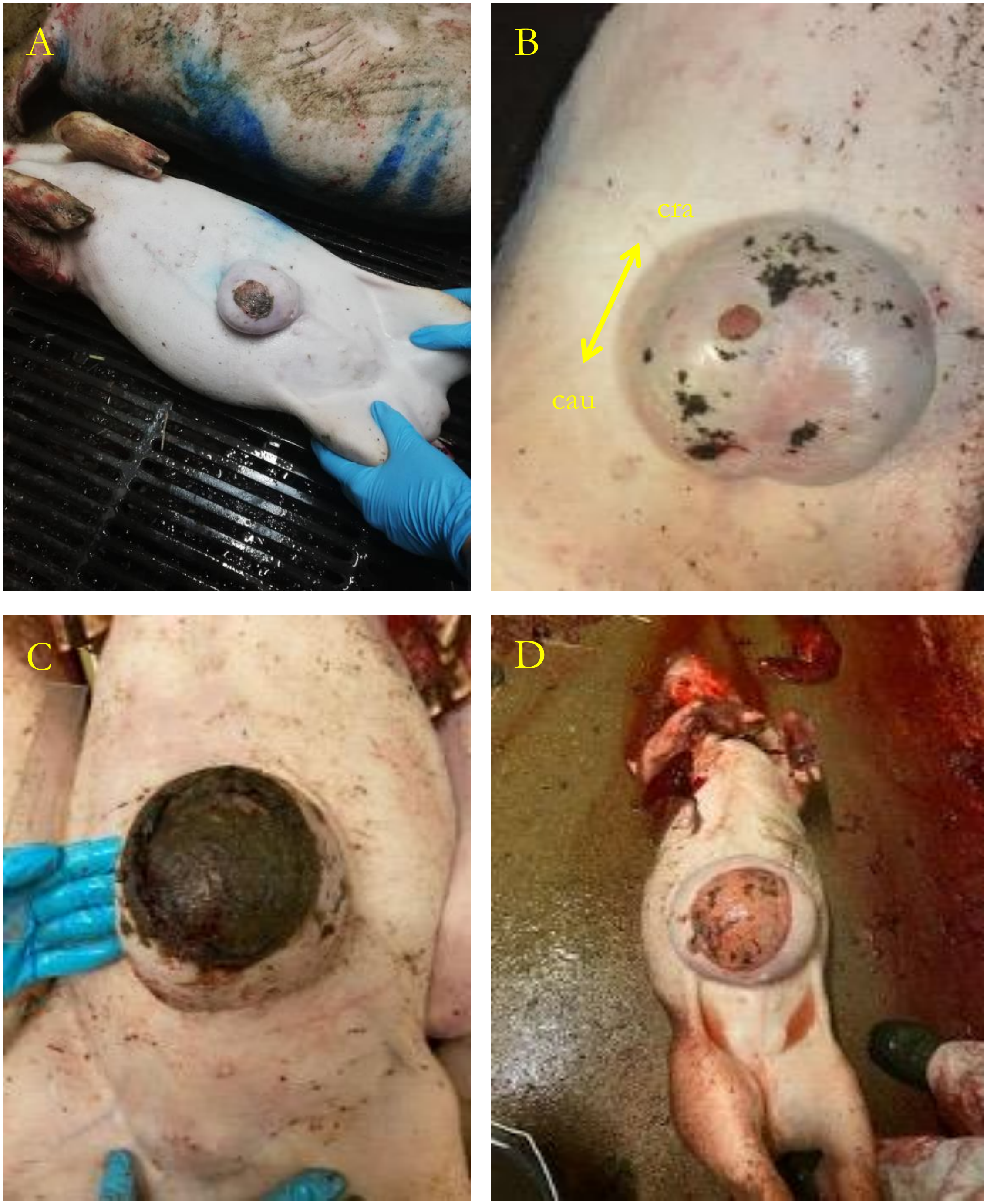

Pig	Weight	Size UO	Size ulcer
A	10-20 kg	7x6 cm	3x3 cm
B	> 20 kg	11x10 cm	1x2 cm
C	> 20 kg	13x12 cm	8x9 cm
D	> 20 kg	17x17 cm	14x12 cm

Notice deep ulcer and lots of manure sticking to wound surface of pig C.

Types of umbilical outpouchings

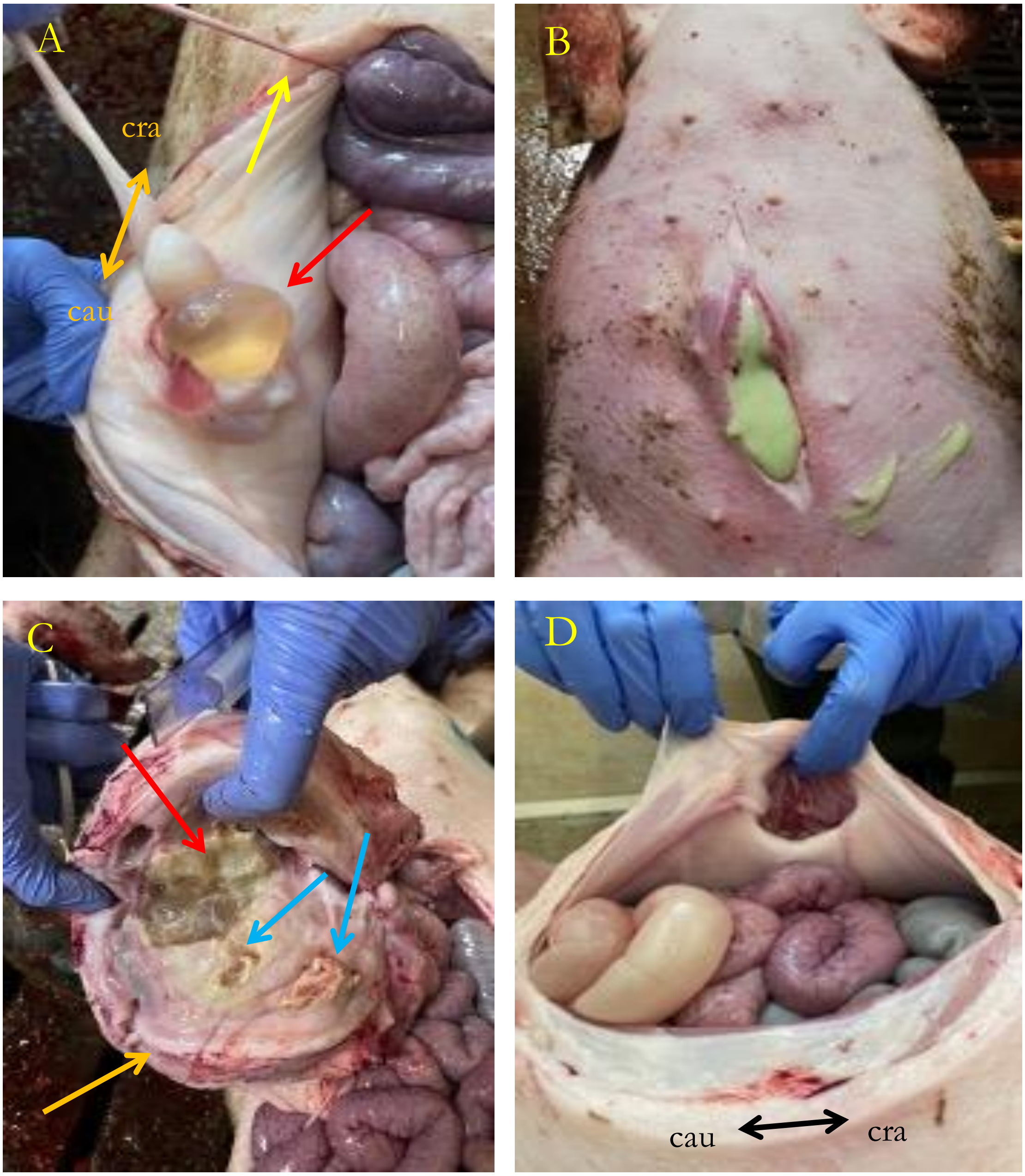

Pig	Weight	Pathological findings
A	10-20 kg	Cyst (red arrow)
		Lig. teres hepatis = round liver ligament (yellow arrow)
B	> 20 kg	Abscess, liquid green pus
C	10- 20 kg	*Combined* diagnose with
		Cyst (red arrow)
		Abscess (blue arrows, cottage cheese like)
		Connective tissue 25 mm (orange arrow)
D	> 20 kg	Hernia with *anulus umbilicus* = umbilical ring

Incarceration and diverticula

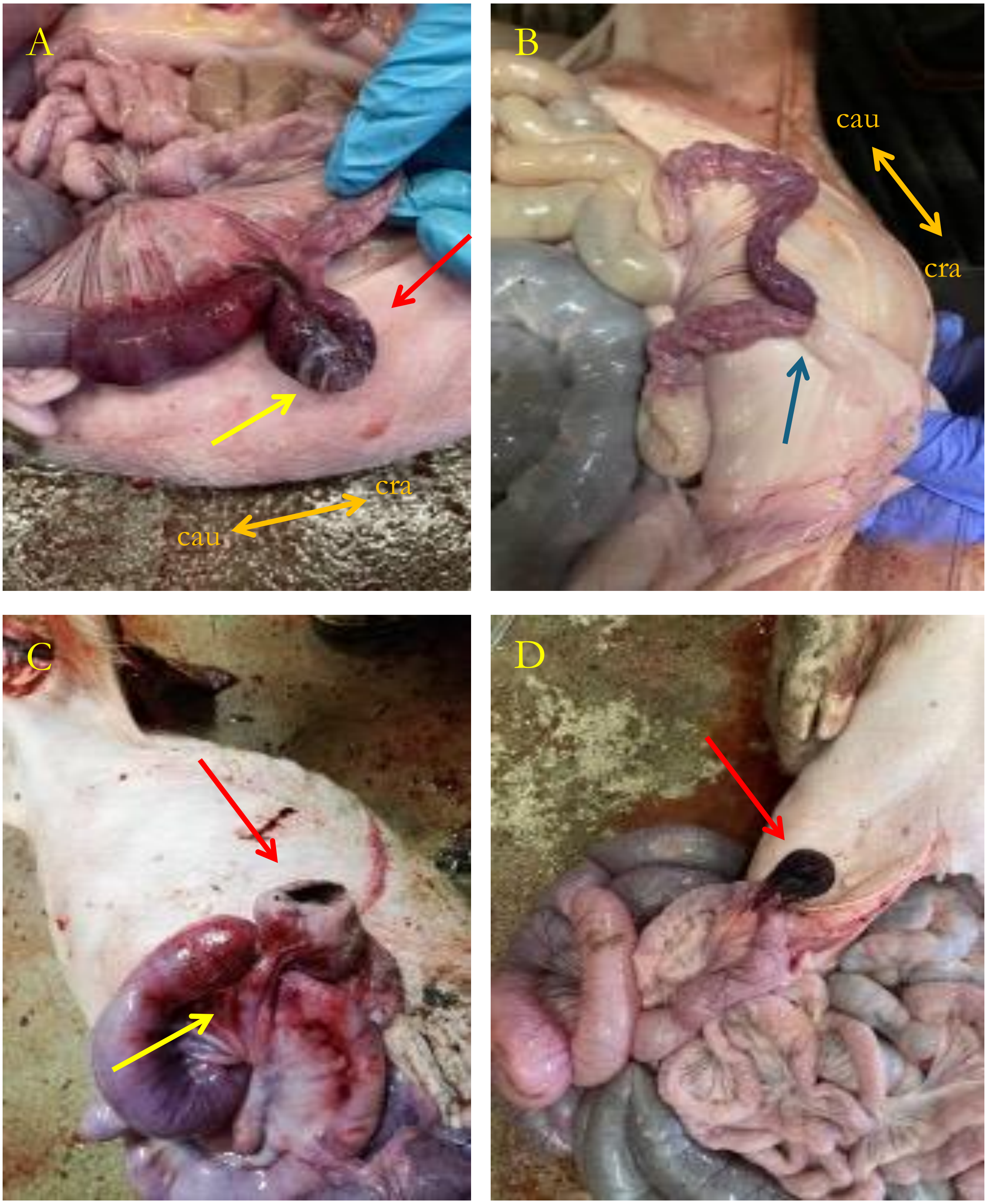

Pig	Weight	Pathological findings
A	> 20 kg	Diverticula ileum (red arrow)
		Hyperaemic intestine after incarceration
		Fibrinous exudate upon the surface of intestine (yellow arrow)
B	> 20 kg	Intestinal loop caught in umbilical ring (yellow arrow)
		with resulting stasis and hyperaemia
C	< 10 kg	Opened diverticula ileum (red arrow)
		Haemorrhagic intestine (yellow arrow
D	> 20 kg	Diverticula jejunum (red arrow) with stasis and hyperaemia
		after incarceration in umbilical ring

Bleeding and adhesions

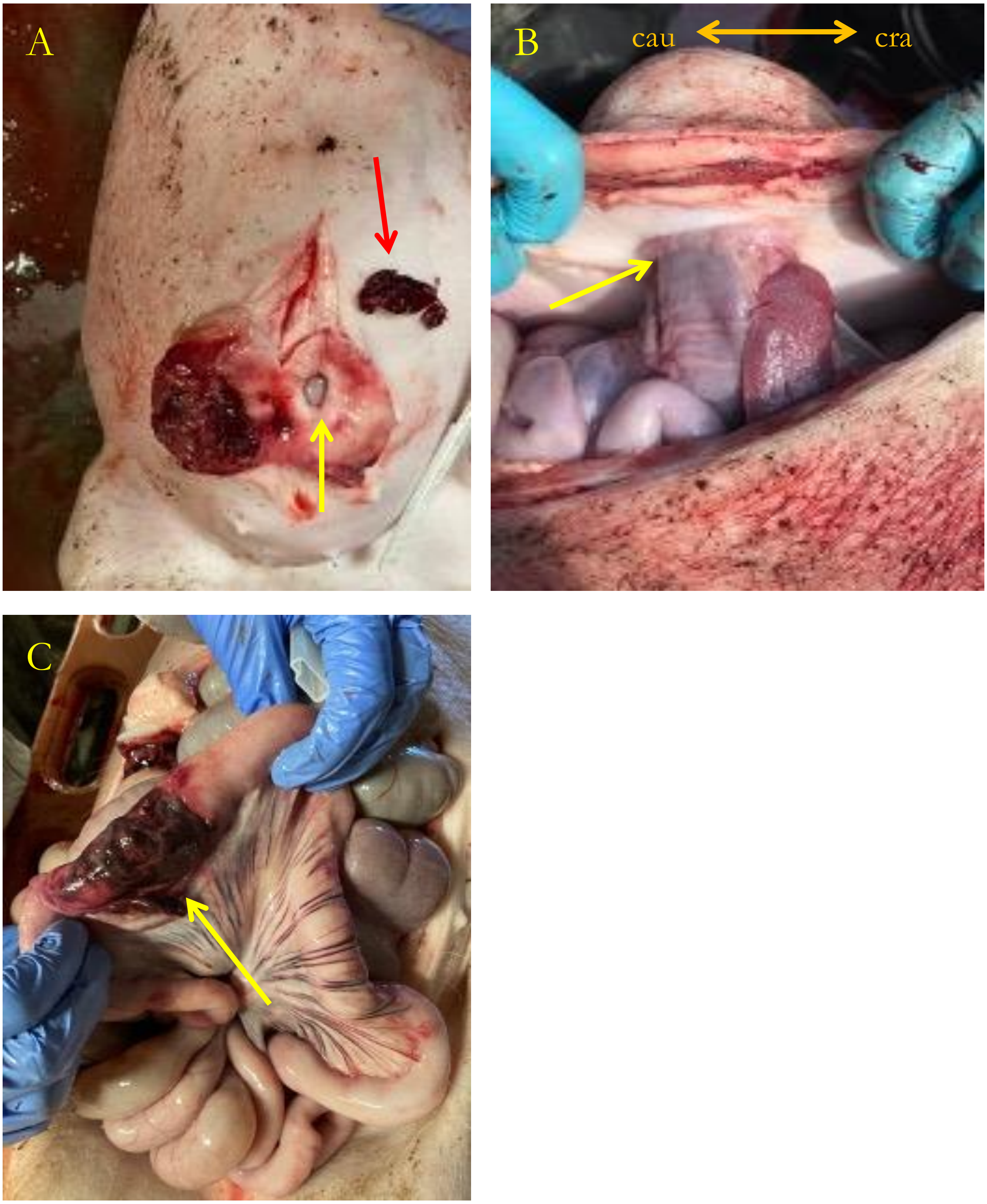

Pig	Weight	Pathological findings
A	20 kg	Bleeding hernial sack<br>Haematoma (red arrow)<br>*Anulus umbilicalis* = umbilical ring (yellow arrow)
B	> 20 kg	Adhesions between umbilical ring, intestines, and spleen (yellow arrow)
C	> 20 kg	Intestinal haemorrhage (yellow arrow)

Umbilical ligaments

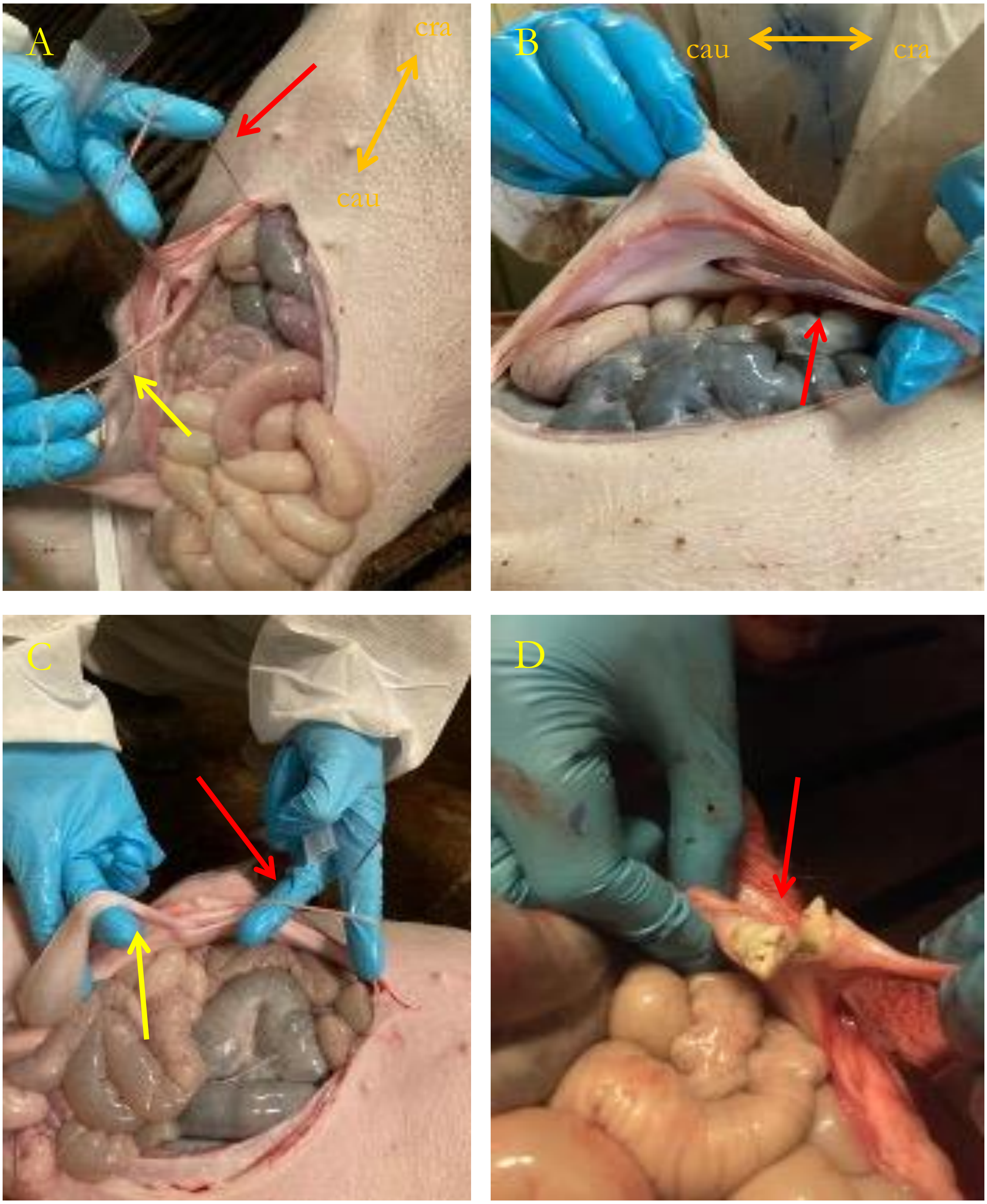

Pig	Weight	Pathological findings
A	10-20 kg	Normal umbilical ligaments,<br>Lig. umbilicale medianum (yellow arrow) &<br>Lig. teres hepatis (red arrow)
B	10-20 kg	Enlarged lig. teres hepatis with signs of inflammation (red arrow)<br>→ Omphalophlebit
C	< 10 kg	Enlarged lig. umbilicale medianum (yellow arrow)<br>(urachus has not regressed and turned into ligament yet)<br>Normal lig. teres hepatis (red arrow)
D	> 20 kg	Abscess urachus (yellow arrow)<br>→ Urachitis

Persistent urachus

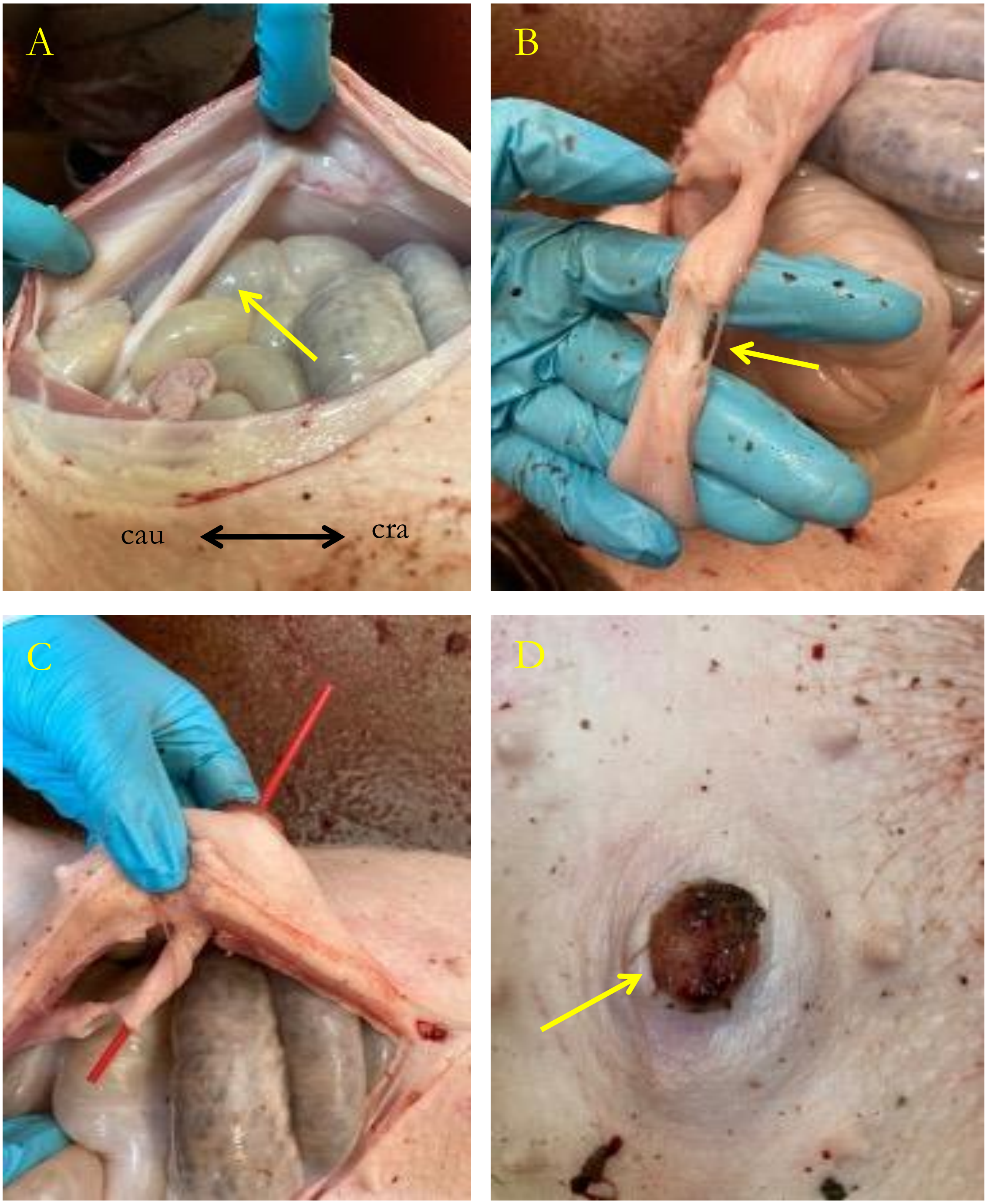

**Pig**	**Weight**	**Pathological findings**
A	10-20 kg	Persistent urachus
B		Lumen urachus
C		Communication from bladder to umbilicus
D		Urachus opens through this ulcer on the abdomen

All pictures originate from one pig
